# p53 protein accumulation predicts resistance to endocrine therapy and decreased post-relapse survival in metastatic breast cancer

**DOI:** 10.1186/bcr1536

**Published:** 2006-07-25

**Authors:** Hiroko Yamashita, Tatsuya Toyama, Mariko Nishio, Yoshiaki Ando, Maho Hamaguchi, Zhenhuan Zhang, Shunzo Kobayashi, Yoshitaka Fujii, Hirotaka Iwase

**Affiliations:** 1 Oncology and Immunology, Nagoya City University Graduate School of Medical Sciences, Kawasumi 1, Mizuho-ku, Nagoya 467-8601, Japan; 2 Breast and Endocrine Surgery, Kumamoto University, 1-1-1 Honjo, Kumamoto 860-8556, Japan

## Abstract

**Introduction:**

Endocrine therapy is the most important treatment option for women with hormone receptor-positive breast cancer. The potential mechanisms for endocrine resistance involve estrogen receptor (ER)-coregulatory proteins and cross-talk between ER and other growth factor-signaling networks. However, the factors and pathways responsible for endocrine resistance are still poorly identified.

**Materials and methods:**

The expression of HER2, p53, and Ki67 was examined by immunohistochemistry in primary breast tumour specimens from 73 metastatic breast cancer patients who received first-line treatment with endocrine therapy on relapse, and analysed to determine whether expression of these molecular markers affected the response to endocrine therapy.

**Results:**

Of the 73 invasive ductal carcinomas, 12.3%, 21.9%, and 35.6% were positive for HER2 overexpression, p53 protein accumulation, and Ki67 expression, respectively. All patients received endocrine therapy as first-line treatment for metastatic breast cancer; 34 patients (46.6%) responded. Patients with primary breast tumours that had p53 protein accumulation and Ki67 expression showed significantly more resistance to endocrine therapy (*P *= 0.0049 and *P *= 0.024, respectively). There were also tendencies for HER2 overexpression to correlate with resistance to endocrine therapy, but this did not reach significance. p53 protein accumulation and HER2 overexpression significantly reduced post-relapse survival (*P *< 0.0001 and *P *= 0.001, respectively), and these factors were also statistically significant in a multivariate analysis.

**Conclusion:**

These data suggest that p53 protein accumulation is helpful in selecting patients who may benefit from endocrine therapy and is a prognostic marker in hormone receptor-positive metastatic breast cancer.

## Introduction

Endocrine therapy has become the most important treatment option for women with estrogen receptor (ER)-positive breast cancer. Nevertheless, many breast cancer patients with tumours expressing high levels of ER are unresponsive to endocrine therapy, and all patients with advanced disease eventually develop resistance to the therapy. The potential mechanisms behind either intrinsic or acquired endocrine resistance involve ER-coregulatory proteins and cross-talk between the ER pathway and other growth factor-signaling networks [1,2]. An understanding of the molecular mechanisms that modulate the activity of the estrogen-signaling network has enabled new ways of overcoming endocrine resistance to be developed.

Several specific proteins have been suggested to contribute to endocrine therapy resistance. HER2 overexpression might be associated with reduced efficacy of adjuvant endocrine therapy with tamoxifen [3-5]. De Laurentiis and colleagues recently conducted a meta-analysis and found that HER2-positive metastatic breast cancer was less responsive to any type of endocrine therapy [6].

Nearly one third of breast tumours have mutations in the *p53 *gene, which are associated with high histological grade and rapid progression [7]. Immunohistochemical assays generally detect nuclear accumulation of the protein, which is often related to conformational alterations and a prolonged half-life of the encoded protein [8,9]. Mutation of the *p53 *gene or overexpression of its protein product has been identified in 14% to 52% of primary breast tumour specimens, and these alterations were found to be associated with poor prognosis in an analysis of more than 3,000 patients with primary breast cancer [10]. We recently analysed the expression of HER2, p53, and Ki67 in 506 invasive ductal carcinoma tissues and showed that the coexistence of HER2 overexpression and p53 protein accumulation was a strong prognostic molecular marker in breast cancer [11].

Apoptosis may represent a common mechanism whereby a variety of antitumour treatments results in cell death. Cells with loss of *p53 *gene function may be unable to undergo apoptosis and thus be resistant to these forms of therapy [12]. Because mutated protein might accumulate in cells, immunohistochemical staining has been a popular surrogate marker for *p53 *mutational status. Studies have been published that examined the predictive value of *p53 *alterations at the gene level, or p53 expression status by immunohistochemistry (IHC), for response to chemotherapy. Many of these studies reported that p53 alterations predict resistance to anthracyclins [13-19]; cyclophosphamide, methotrexate, and fluorouracil (CMF); or other agents [20-22]. However, of the few studies that have examined the association between p53 status and response to endocrine therapy [23-27], one study [27] reported a value for p53 in predicting endocrine resistance.

We previously described that expression of ER-α and progesterone receptor (PR) by IHC in primary breast tumours is predictive of response to endocrine therapy in patients with metastatic breast cancer who received endocrine therapy on relapse [28]. In this study, we examined the expression of HER2, p53, and Ki67 by IHC in primary breast tumour specimens from 73 patients with metastatic breast cancer who received first-line treatment with endocrine therapy on relapse, and analysed whether expression of these molecular markers affected the response to endocrine therapy.

## Materials and methods

### Patients and breast cancer tissues

Breast tumour specimens from 73 female patients with metastatic breast cancer, who were treated at Nagoya City University Hospital (Nagoya, Japan) between 1982 and 2002, were included in this study (Table 1). The study protocol was approved by the institutional review board and conformed with the guidelines of the 1975 Declaration of Helsinki. All patients had undergone surgical treatment for primary breast cancer (either mastectomy or lumpectomy), and all primary tumours were ER- or PR-positive. After surgery, five patients (6.9%) received no additional therapy. Of the remaining 68 patients, 32 (43.8%) received systemic adjuvant therapy consisting of endocrine therapy (tamoxifen) alone, two (2.7%) received chemotherapy alone, and 34 (46.6%) received combined endocrine therapy and chemotherapy. Patients who were positive for axillary lymph node metastases received either oral administration of 5-fluorouracil derivatives for 2 years or a combination of cyclophosphamide, methotrexate, and fluorouracil (CMF). Patients were observed for disease recurrence at least once every 6 months for the first 5 years after the surgery and thereafter once every year.

### First-line endocrine therapy for metastatic breast cancer and response criteria

When the patients relapsed and were diagnosed with metastatic breast cancer, they started endocrine therapy (Table 1). Patients were assessed monthly for clinical response, which was defined according to World Health Organization criteria as complete response, partial response, no change, and progressive disease. The presence of progressive disease indicated treatment failure; all other clinical responses were considered to show efficacy of treatment. Stable disease was included as a response to treatment because patients with stable disease clearly benefited clinically in metastatic breast cancer.

### Immunohistochemical analysis for ER-α, PR, HER2, p53, and Ki67

One 4-μm section of each submitted paraffin block was first stained with hematoxylin and eosin to verify that an adequate number of invasive ductal carcinoma cells were present, and the fixation quality was sufficient for immunohistochemical analysis as described previously [11,28]. Serial sections (4-μm) were prepared from selected blocks and float-mounted on adhesive-coated glass slides for ER-α, PR, HER2, p53, or Ki67 staining. Primary antibodies included monoclonal mouse anti-human ER antibody (1D5; Dako Denmark A/S, Glostrup, Denmark) at 1:100 dilution for ER-α, monoclonal mouse anti-human PgR antibody (636; Dako Denmark A/S) at 1:100 dilution for PR, rabbit anti-human c-erbB-2 oncoprotein antibody (Dako Denmark A/S) at 1:200 dilution for HER2, monoclonal mouse anti-human p53 protein antibody (PAb1801; Novocastra, Newcastle, UK) at 1:50 dilution for p53, and monoclonal mouse anti-human Ki67 antibody (MIB-1; Dako Denmark A/S) at 1:100 dilution for Ki67. The Dako Denmark A/S EnVision system (Dako Denmark A/S EnVision labeled polymer, peroxidase) was used as the detection system for ER-α, PR, HER2, and Ki67. The streptavidin-biotin system (SAB-PO kit; Nichirei Co., Inc., Tokyo, Japan) was used for detection of the bound antibody of p53.

### Immunohistochemical scoring

Immunostained slides were scored after the entire slide was evaluated by light microscopy. The expression of ER-α and PR was scored by assigning proportion and intensity scores, in accordance with the procedure of Allred and colleagues [29]. Any brown nuclear staining in invasive breast epithelium was counted toward the proportion score. Tumours with a score of 3 or greater were considered to be positive for ER-α and PR expression. HER2 immunostaining was evaluated by the same method as the HercepTest (Dako Denmark A/S). To determine the score of HER2 expression, the membrane staining pattern was estimated and scored on a scale of 0 to 3. Tumours with a score of 3 were considered to be positive for HER2 overexpression. The expression status of p53 and Ki67 was assessed according to the estimated proportion of nuclear staining of tumour cells that were positively stained as described previously [11]. Scoring criteria were as follows: for p53, score of 0 = none; score of 1, <1/10; score of 2, 1/10 to 1/2; and score of 3, >1/2; for Ki67, score of 0 = none; score of 1, <1/100; score of 2, 1/100 to 1/10; score of 3, 1/10 to 1/2; and score of 4, >1/2. Tumours with a score of 2 or greater for p53 were considered to be positive for p53 protein accumulation, and tumours with a score of 3 or greater for Ki67 were considered to be positive for Ki67 expression.

### Statistical analysis

The χ^2 ^test was used to compare the immunohistochemical results of molecular markers with clinicopathological characteristics and response to endocrine therapy. Estimation of post-relapse survival was performed using the Kaplan-Meier method, and differences between survival curves were assessed with the log-rank test. Cox's proportional hazards model was used for univariate and multivariate analyses of prognostic values.

## Results

### Correlation between HER2, p53, and Ki67 expression and clinicopathological factors

**Figure 1:**
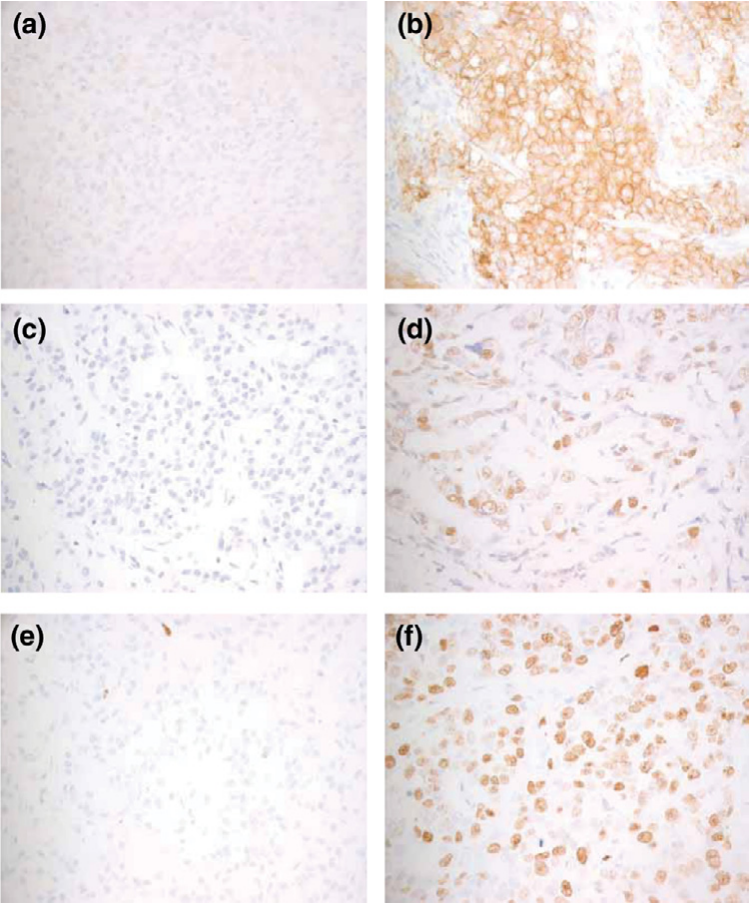
Representative immunohistochemical staining of HER2, p53, and Ki67 in invasive ductal carcinoma. (a) Negative staining of HER2. (b) Positive staining of HER2. (c) Negative staining of p53. (d) Positive staining of p53. (e) Negative staining of Ki67. (f) Positive staining of Ki67.

We examined expression of HER2, p53, and Ki67 in primary invasive breast carcinomas by IHC from 73 patients with metastatic breast cancer who received endocrine therapy as first-line treatment on relapse (Figure 1). The immunohistochemical status for HER2, p53, and Ki67 was compared among patient subgroups according to clinicopathological factors. Of the 73 invasive ductal carcinomas, 12.3%, 21.9%, and 35.6% were positive for HER2 overexpression, p53 protein accumulation, and Ki67 expression, respectively (Table 2). No association was found between HER2 overexpression, p53 protein accumulation, or Ki67 expression and clinicopathological factors.

### p53 protein accumulation and Ki67 expression in primary breast tumours are predictive of endocrine therapy resistance in metastatic breast cancer

At relapse, all patients received endocrine therapy as first-line treatment for metastatic breast cancer; 34 patients (46.6%) responded. We analysed whether the expression status of HER2, p53, and Ki67 in the primary breast tumours affected the response to endocrine therapy in this setting (Table 3). Patients with primary breast tumours that had p53 protein accumulation and Ki67 expression were significantly more likely to exhibit resistance to endocrine therapy (*P *= 0.0049 and *P *= 0.024, respectively). Patients with HER2 overexpression also showed a tendency for greater resistance to endocrine therapy (*P *= 0.054), but the trend did not reach statistical significance.

### Patients with HER2 overexpression and p53 protein accumulation in primary breast tumours had a significantly shorter survival after relapse

**Figure 2:**
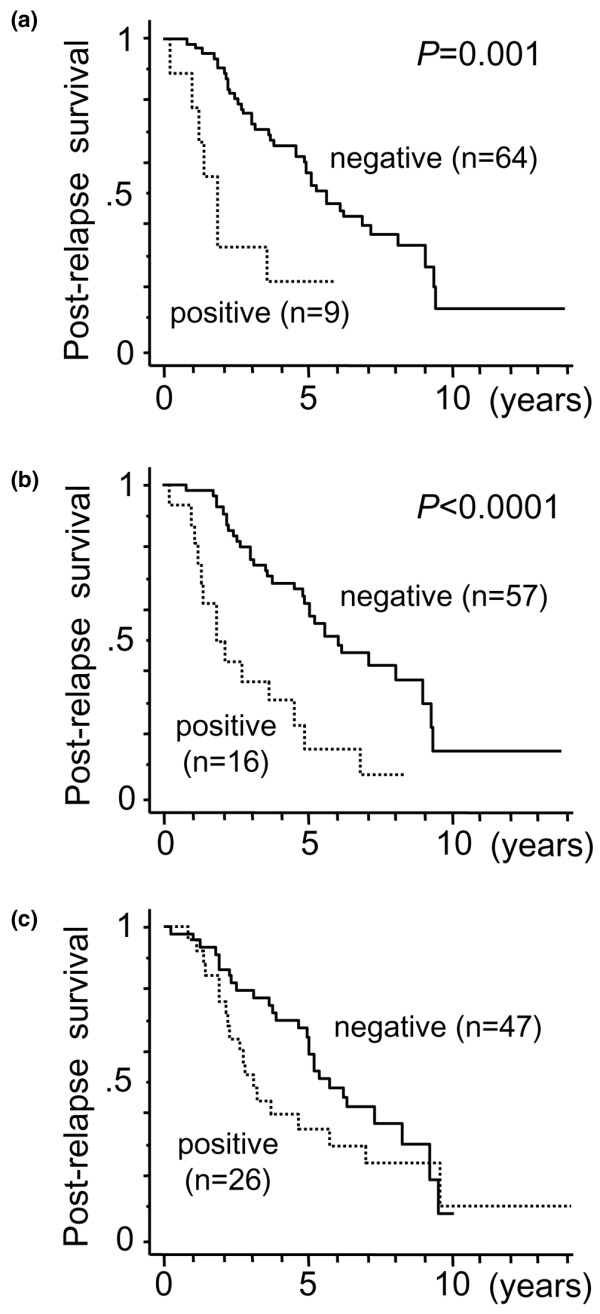
Post-relapse survival according to HER2 overexpression, p53 protein accumulation, and Ki67 expression. (a) Post-relapse survival according to HER2 overexpression. (b) Post-relapse survival according to p53 protein accumulation. (c) Post-relapse survival according to Ki67. HER2 overexpression and p53 protein accumulation significantly reduced post-relapse survival.

We analysed whether expression status of HER2, p53, and Ki67 in the primary breast tumours affected survival after relapse. The median follow-up period was 77 months (range, 4 to 234 months). HER2 overexpression significantly reduced post-relapse survival (*P *= 0.001) (Figure 2a) by log-rank test. Moreover, patients with p53 protein accumulation had a significantly shorter post-relapse survival (*P *< 0.0001) (Figure 2b). Univariate analysis (Table 4) demonstrated significant associations between post-relapse survival and HER2 overexpression (*P *= 0.0024) and p53 protein accumulation (*P *= 0.0002) as well as expression ER-α (*P *= 0.0009) and PR (*P *= 0.0012) expression. On the other hand, Ki67 expression did not affect post-relapse survival. The status of expressions of ER-α, PR, HER2, and p53 were selected for the multivariate analysis. Patients with primary tumours with HER2 overexpression (*P *= 0.0046) and p53 protein accumulation (*P *= 0.013) had significantly reduced post-relapse survival, whereas those with positive expression of PR had significantly increased post-relapse survival (*P *= 0.045) (Table 4). We conclude that HER2 overexpression and p53 protein accumulation are independent prognostic factors of post-relapse survival in patients with metastatic breast cancer who received first-line treatment with endocrine therapy on relapse.

## Discussion

The present study indicates that p53 protein accumulation predicts resistance to endocrine therapy and decreased post-relapse survival in patients with metastatic breast cancer who received first-line treatment with endocrine therapy on relapse.

Experimental data suggest a complex cross-talk between HER2 and ER, and it has been hypothesised that HER2-positive tumours may be less responsive to certain endocrine treatments. However, it has yet to be established whether HER2 overexpression is predictive of resistance to endocrine therapy, whether used as an adjuvant therapy after excision of localised breast cancer or as treatment for metastatic disease. In the metastatic setting, several groups have identified a correlation between HER2 overexpression and a lower response rate to endocrine therapy, whereas others have not. De Laurentiis and colleagues recently conducted a meta-analysis of the published studies to obtain an overall pooled estimate of the association between HER2 overexpression and treatment failure rate [6]. They found that HER2-positive metastatic breast cancer was less responsive to any type of endocrine therapy. Our data failed to show an association between HER2 overexpression and resistance to endocrine therapy, possibly due to the small number of patients in our study. Nevertheless, HER2 overexpression significantly reduced post-relapse survival.

It has been reported from three groups of clinical trials that p53 status by IHC does not predict response to tamoxifen [23-25]. Elledge *et al*. found that p53 expression as measured by IHC was not associated with response to tamoxifen in 205 patients with metastatic breast cancer, although patients with higher p53 had a worse survival [25]. It has also been reported not to predict tamoxifen resistance in ER-positive, node-positive breast cancer [24] or in high-risk postmenopausal breast cancer [23] in adjuvant studies. In contrast, Berns *et al*. found that in 401 patients with metastatic breast cancer, p53 expression as measured by enzyme immunoassay of cytoplasmic extracts from primary tumours was a predictor of tamoxifen resistance [27]. Our results indicate that p53 protein accumulation by IHC was predictive of endocrine therapy resistance and decreased post-relapse survival in metastatic breast cancer. The antibody for p53 that Elledge *et al*. [25] used was also used in our study. Their results may have differed from ours because not only tamoxifen, but also aromatase inhibitors and/or LHRH (luteinising hormone-releasing hormone) agonists, were used as endocrine therapy in our study. Further studies to analyse the correlation between response to each endocrine therapy agent and p53 status are necessary to understand the role of p53 in endocrine therapy resistance. Moreover, the conflicting results may be caused by the different evaluation methods of p53 status. Geisler *et al*. demonstrated that nearly 30% of all mutations in primary breast cancer, in particular those of the nonsense type, are not detected by IHC and that most studies evaluating p53 status by IHC have failed to show a predictive value of p53 for chemotherapy, whereas studies evaluating p53 status by DNA sequencing have reported mutations that predict resistance to chemotherapy [15]. Therefore, mutational analysis of p53 might be helpful in evaluating the association between p53 status and endocrine therapy resistance.

## Conclusion

The present study indicates that p53 protein accumulation predicts resistance to endocrine therapy and decreased post-relapse survival in patients with metastatic breast cancer who received first-line treatment with endocrine therapy on relapse. Our data suggest that p53 protein accumulation is helpful in selecting patients who may benefit from endocrine therapy and is a prognostic marker in metastatic breast cancer.

## Abbreviations

CMF = cyclophosphamide, methotrexate, and fluorouracil; ER = estrogen receptor; IHC = immunohistochemistry; PR = progesterone receptor.

## Competing interests

The authors declare that they have no competing interests.

## Authors' contributions

HY conceived of the study and participated in its design, coordination, and manuscript writing. MN carried out immunostaining experiments. SK, YF, and HI participated in its design and coordination and helped to draft the manuscript. TT, YA, and MH provided tissue samples. HY and ZZ assessed the immunostaining. All authors read and approved the final manuscript.

## Figures and Tables

**Table 1:** Clinicopathological characteristics of patients, primary breast tumours, and treatment

		Number of patients (percentage)
Total number of patients		73
Age at diagnosis (years)	≤50 >50	34 (46.6) 39 (53.4)
	Range	29 to 77
Tumour size (cm)	<2.0 ≥2.0	20 (27.4) 53 (72.6)
Number of positive lymph nodes	0 1–3 >3	21 (28.8) 20 (27.4) 32 (43.8)
Histological grade	1 2 3	12 (16.4) 42 (57.5) 19 (26.1)
Adjuvant therapy	None Endocrine therapy Chemotherapy Combined	5 (6.9) 32 (43.8) 2 (2.7) 34 (46.6)
Disease-free interval (months)	Mean ± SD Median	39.8 ± 25.7 38
	Range	2 to 123
First-line endocrine therapy for metastatic breast cancer	Tamoxifen Aromatase inhibitors LHRH agonist LHRH agonist + tamoxifen Fulvestrant	56 (76.7) 9 (12.3) 3 (4.1) 4 (5.5) 1 (1.4)

LHRH, luteinising hormone-releasing hormone; SD, standard deviation.

**Table 2:** Correlation between clinicopathological factors and molecular markers

	HER2		P53		Ki67	
	
	Positive/total (percentage)	*P*	Positive/total (percentage)	*P*	Positive/total (percentage)	*P*
Total	9/73 (12.3)		16/73 (21.9)		26/73 (35.6)	
						
Tumour size (cm)						
<2.0	4/20 (20.0)	0.41	5/20 (25.0)	0.94	7/20 (35.0)	>0.99
≥2.0	5/53 (9.4)		11/53 (20.8)		19/53 (35.8)	
						
Number of positive lymph nodes						
0	1/22 (4.5)	0.34	3/22 (12.6)	0.41	6/22 (27.2)	0.47
≥1	8/51 (15.7)		13/51 (25.5)		20/51 (39.2)	
						
Histological grade						
1	0/12 (0)	0.36	0/12 (0)	0.05	3/12 (25.0)	0.18
2	6/42 (14.3)		9/42 (21.4)		13/42 (31.0)	
3	3/19 (15.8)		7/19 (36.8)		10/19 (52.6)	
						
Estrogen receptor-α						
Negative	4/17 (23.5)	0.23	5/17 (29.4)	0.60	8/17 (47.1)	0.40
Positive	5/56 (8.9)		11/56 (19.6)		18/56 (32.1)	
Progesterone receptor						
Negative	3/18 (16.7)	0.81	5/18 (27.8)	0.71	9/18 (50.0)	0.23
Positive	6/55 (10.9)		11/55 (20.0)		17/55 (30.9)	

**Table 3:** Correlation between immunohistochemical status of HER2, p53, and Ki67 and response to endocrine therapy

	HER2		P53		Ki67	
	
	Positive/total (percentage)	*P*	Positive/total (percentage)	*P*	Positive/total (percentage)	*P*
Response to endocrine therapy (*n *= 73)						
Total	9/73 (12.3)		16/73 (21.9)		26/73 (35.6)	
Responders	1/34 (2.9)	0.054	2/34 (5.9)	0.0049^a^	7/34 (20.6)	0.024^a^
Non-responders	8/39 (20.5)		14/39 (35.9)		19/39 (48.7)	

^a^*P *< 0.05 is considered significant.

**Table 4:** Prognostic factors in 73 invasive ductal carcinomas compared with post-relapse survival

	Univariate			Multivariate		
	*P*	RR	95% CI	*P*	RR	95% CI

Age	0.67	0.882	0.494–1.576			
Tumour size	0.21	1.483	0.797–2.759			
Lymph node status	0.40	0.746	0.376–1.479			
Histological grade	0.15	0.500	0.197–1.271			
ER-α	0.0009^a^	0.342	0.181–0.645	0.79	1.174	0.354–3.886
PR	0.0012^a^	0.358	0.192–0.668	0.045^a^	0.291	0.087–0.972
HER2	0.0024^a^	3.637	1.582–8.362	0.0046^a^	3.710	1.498–9.191
p53	0.0002^a^	3.371	1.765–6.440	0.013^a^	2.364	1.198–4.665
Ki67	0.16	1.539	0.849–2.790			

^a^*P *< 0.05 is considered significant. CI, confidence interval; ER, estrogen receptor; PR, progesterone receptor; RR, relative risk.
